# Nociceptive behavioural assessments in mouse models of temporomandibular joint disorders

**DOI:** 10.1038/s41368-020-00095-0

**Published:** 2020-09-29

**Authors:** Jun Li, Kaige Ma, Dan Yi, Chun-do Oh, Di Chen

**Affiliations:** 1 Department of Orthopedic Surgery, Rush University Medical Center, Chicago, IL 60612 USA; 2 Research Center for Human Tissues and Organs Degeneration, Shenzhen Institutes of Advanced Technology, Chinese Academy of Sciences, Shenzhen, 518055 China

**Keywords:** Cell biology, Diseases

## Abstract

Orofacial pain or tenderness is a primary symptom associated with temporomandibular joint (TMJ) disorders (TMDs). To understand the pathological mechanisms underlying TMDs, several mouse models have been developed, including mechanical stimulus-induced TMD and genetic mouse models. However, a lack of feasible approaches for assessing TMD-related nociceptive behaviours in the orofacial region of mice has hindered the in-depth study of TMD-associated mechanisms. This study aimed to explore modifications of three existing methods to analyse nociceptive behaviours using two TMD mouse models: (1) mechanical allodynia was tested using von Frey filaments in the mouse TMJ region by placing mice in specially designed chambers; (2) bite force was measured using the Economical Load and Force (ELF) system; and (3) spontaneous feeding behaviour tests, including eating duration and frequency, were analysed using the Laboratory Animal Behaviour Observation Registration and Analysis System (LABORAS). We successfully assessed changes in nociceptive behaviours in two TMD mouse models, a unilateral anterior crossbite (UAC)-induced TMD mouse model and a β-catenin conditional activation mouse model. We found that the UAC model and β-catenin conditional activation mouse model were significantly associated with signs of increased mechanical allodynia, lower bite force, and decreased spontaneous feeding behaviour, indicating manifestations of TMD. These behavioural changes were consistent with the cartilage degradation phenotype observed in these mouse models. Our studies have shown reliable methods to analyse nociceptive behaviours in mice and may indicate that these methods are valid to assess signs of TMD in mice.

## Introduction

The temporomandibular joint (TMJ) is one of the most frequently used joints in the human body,^1^ and TMJ disorders (TMDs) are commonly encountered in dental clinical practice, with a prevalence of 4%–10% worldwide.^2,3^ TMD patients present with at least one of the following symptoms: pain, mandibular mobility limitations, TMJ noise, and clicking. Orofacial pain is a primary clinical complaint reported by patients who seek treatment.^4,5^ Although several mouse models have been developed to explore the pathological mechanisms underlying TMDs, few feasible approaches for assessing nociceptive behaviours in the orofacial region have been developed in mice. Behavioural alterations, especially nociceptive phenotypes, are key clinical indicators for determining the similarity between animal models and human TMDs and are important for evaluating the efficacy of drug therapy.

Despite the challenges faced when assessing behavioural responses in the orofacial region, due to the location and complex anatomy of the TMJ, several attempts have been made to develop direct behavioural tests.^6,7^ Ren reported, for the first time in 1999, a method for assessing mechanical allodynia in the rat TMJ using a monofilament.^7^ Subsequently, this method has been improved in several other studies and is currently performed by placing a rat into an individual plastic cage and measuring the response threshold using a digital device consisting of a rigid filament linked to an electronic device.^8–12^ However, the more disobedient and aggressive temperament of mice compared with rats has complicated the development of a similar mechanical allodynia measurement method for the mouse orofacial region. Therefore, improving nociceptive testing methods for TMD mouse models remains an essential goal.

A dominant clinical feature of TMDs is pain associated with chewing or mastication. Recent studies have suggested that the direct measurement of bite force may represent a novel approach for the quantification of TMD-associated mastication-related nociceptive behaviours in mouse models.^13,14^ In addition, feeding behaviours are associated with mastication and could provide another critical indicator for the study of TMD-associated nociceptive behaviours.^7,15^ Altered eating patterns, including changes in food intake, eating duration, and eating frequency, could reflect changes in masticatory function caused by TMDs. Recent studies demonstrated that TMDs may also affect other tissues in addition to the TMJ, such as orofacial muscles and nerves, which could also lead to mastication dysfunction.^16–20^ Therefore, the bite force test and other tests evaluating changes in chewing behaviours that might reflect dysfunction of the jaw muscles and periodontal tissues^21–24^ also merit further investigation.

Typical mouse models of TMDs include both mechanical and genetic models. In this study, we utilised one type of mechanical stimulus-induced TMD mouse model and one type of genetic mouse model, both of which have been used and reported in our previous studies.^25,26^ Aberrant biomechanical stimulation has been characterised as a critical factor that induces cartilage degradation in the TMJ.^27^ Our previous report indicated that the unilateral anterior crossbite (UAC) prosthesis could cause excessive joint loading and abnormal dental occlusion, leading to the development of degenerative TMJ lesions in mice.^25^ In addition, a genetically modified mouse model is advantageous for TMD research. Our previous study showed that β-catenin conditional activation mice, β-catenin (ex3)^Agc1ER^ mice or β-catenin^Act^ mice, exhibited a phenotype consistent with progressive TMJ disorder.^26^

Therefore, the present study aimed to explore three quantitative approaches for nociceptive behavioural assessments, including a mechanical allodynia test, a bite force test, and spontaneous feeding behavioural assessments, using two TMD mouse models.

## Results

**Fig. 1:**
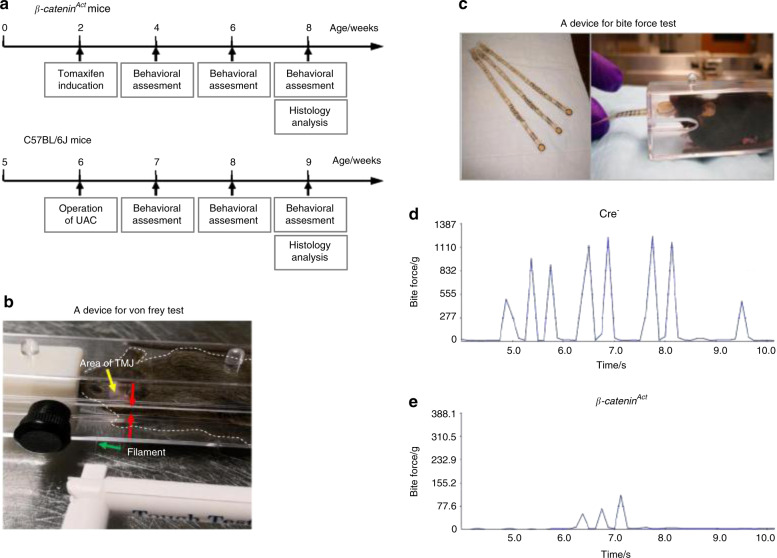
The experimental flow and methods used to assess the mechanical allodynia thresholds and bite forces in mice with TMDs. **a** Experimental flow chart. **b** A mouse was placed into a cage with a gap at the centre (red arrows). The von Frey filament (green arrow) was passed through the gap and probed against the mouse orofacial skin in the TMJ region (yellow arrow). **c** A FlexiForce sensor, from Tekscan (left), was passed through the gap and bit by the mouse (right). **d**, **e** Representative force–time graphs for β-catenin^Act^ and Cre^–^ mice

In this study, we assessed changes in nociceptive behaviours by performing a mechanical allodynia test, a bite force test, and a spontaneous feeding behavioural test using two TMD mouse models. The experimental flow chart is shown in Fig. 1a. The von Frey test (mechanical allodynia test) and the bite force test are shown in Fig. 1b, c. Examples of the bite force test results in Cre^−^ and β-catenin^Act^ mice are presented in Fig. 1d, e.

### UAC induces TMJ cartilage degradation and increases mechanical allodynia

**Fig. 2:**
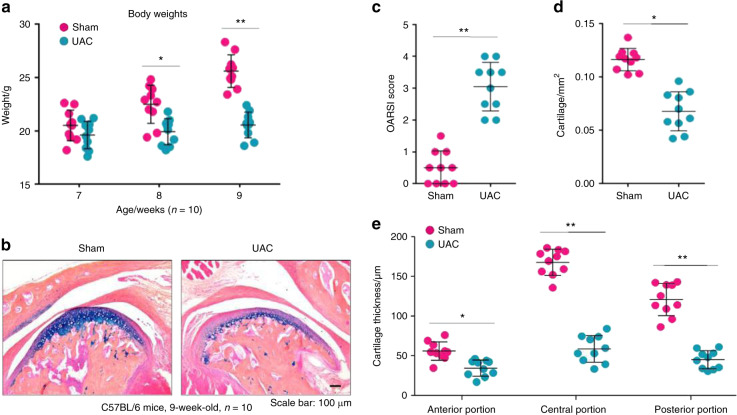
OA-like lesions appeared in the mouse TMJ cartilage in 9-week-old mice (3 weeks after the unilateral anterior crossbite (UAC) operation). **a** Body weights were measured in 7-, 8- and 9-week-old mice (1-, 2-, and 3-weeks post operation) (*n* = 10). **b** Decreased cartilage thickness and chondrocytes with reduced Alcian blue/haematoxylin staining were observed after the operation. **c** The severity of the OA-like phenotype was analysed by assessing the histological grade in the TMJ using the OARSI scoring system (*n* = 10). **d** The mouse condylar cartilage area was quantified by tracing the Alcian blue-positive areas using the OsteoMeasure system (*n* = 10). **e** Quantitative analysis of changes in the mouse condylar cartilage in the anterior, central, and posterior thirds (*n* = 10). Statistical analysis was conducted using an unpaired Student’s *t* test. *P < 0.05, **P < 0.01

After the UAC procedure, the body weights of mice were significantly decreased at 8 and 9 weeks of age (2 and 3 weeks after the UAC operation, respectively) (Fig. 2a), likely due to a reduction in food intake. Histological analysis showed a thinner cartilage layer in mice that underwent the UAC operation, accompanied by reduced Alcian blue staining in the condylar areas (Fig. 2b). We found significantly increased Osteoarthritis Research Society International (OARSI) scores for condylar cartilage damage in 9-week-old mice that received the UAC operation 3 weeks earlier compared with those in mice that underwent a sham operation (Fig. 2c). In addition, the results of histomorphometric analyses showed significant reductions in the cartilage area (Fig. 2d) and thickness (Fig. 2e) in 9-week-old mice that received the UAC operation 3 weeks earlier compared with those in mice that received the sham operation. These results suggested that the UAC procedure could cause TMJ cartilage degeneration, which is consistent with previous observations.^28^

**Fig. 3:**
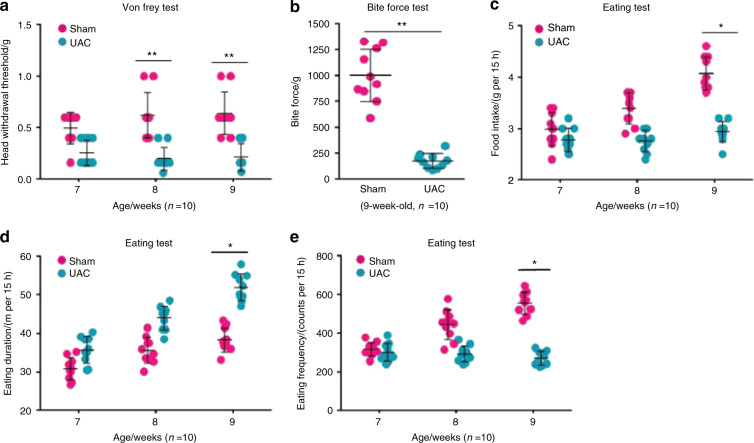
Mechanical allodynia increased in the mouse TMJ region 3 weeks after implanting the UAC prosthesis. **a** The mechanical allodynia threshold was measured using von Frey filaments in mice 1–3 weeks post operation (*n* = 10). **b** The bite force was assessed using FlexiForce sensors in mice 3 weeks post operation when unloading the UAC prosthesis (*n* = 10). **c**–**e** The food intake over 15 h was recorded, and the eating duration and frequency were tested using LABORAS in mice 1–3 weeks post operation (*n* = 10). Statistical analysis was conducted using two-way ANOVA, followed by the Tukey–Kramer post hoc test. *P < 0.05, **P < 0.01

Before the mice were euthanized, we performed a series of behavioural tests to determine whether mechanical allodynia in the TMJ region increased in the mice after the UAC operation. The results from the von Frey test in the 7-, 8-, and 9-week-old mice (1–3 weeks after the UAC operation, respectively) showed that the sensitivity of mechanical allodynia in the TMJ region significantly increased in the mice that underwent the UAC operation compared with in the mice that underwent the sham operation (Fig. 3a). As expected, the bite force was significantly decreased in the mice that underwent the UAC operation compared with the mice that underwent the sham operation (Fig. 3b). The 15-h food intake decreased in the mice that underwent the UAC operation compared with the mice that underwent the sham operation (Fig. 3c). Furthermore, we found that the spontaneous eating duration in the 9-week-old mice (3 weeks after the UAC operation) was significantly extended in the mice that underwent the UAC operation compared with the mice that underwent the sham operation (Fig. 3d). In addition, the eating frequency decreased in the mice that underwent the UAC operation compared with the mice that underwent the sham operation (Fig. 3e). These behavioural assessments suggested that mechanical allodynia significantly increased in the TMJ region of mice after the UAC operation.

### β-catenin activation in mice resulted in a TMJ osteoarthritis (OA) phenotype associated with changes in nociceptive behaviours

**Fig. 4:**
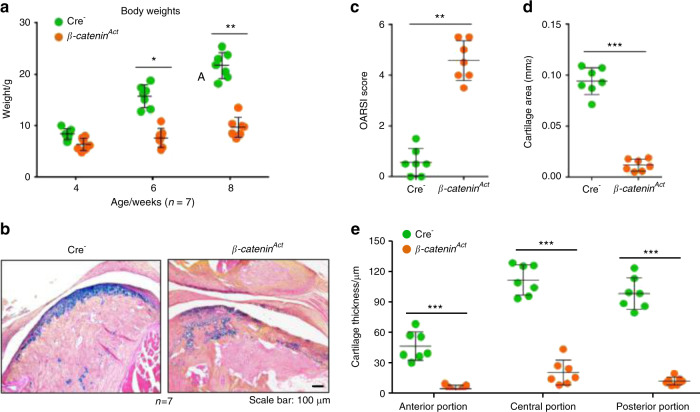
β-catenin conditional activation mice (β-catenin^Act^) develop osteoarthritis (OA)-like phenotypes in the temporomandibular joint (TMJ). **a** Body weights were measured in mice at 4, 6, and 8 weeks of age (*n* = 7). **b** Representative histology images of TMJ tissue, with Alcian blue/haematoxylin staining. **c** The severity of the OA-like phenotype was analysed by assessing the histological grade in the TMJ using the OARSI scoring system (*n* = 7). **d** The mouse condylar cartilage area was quantified by tracing the Alcian blue-positive areas using the OsteoMeasure system (*n* = 7). **e** Quantitative analysis of the changes in the anterior, central, and posterior thirds of the mouse condylar cartilage (*n* = 7). Statistical analysis was conducted using an unpaired Student’s *t* test. *P < 0.05, **P < 0.01, ***P < 0.001

The activation of β-catenin signalling in cartilage tissue caused weight loss in 6- and 8-week-old β-catenin^Act^ mice (Fig. 4a). The results of the histological analyses demonstrated that 8-week-old β-catenin^Act^ mice developed clefts and defects in the condylar cartilage surface, displayed decreased chondrocyte numbers in the superficial and middle layers, and presented with increased numbers of hypertrophic cells in the deep cartilage layer compared with Cre^−^ mice (Fig. 4b). Further analysis of histology sections using the OARSI scoring system showed that β-catenin^Act^ mice presented significantly increased condylar cartilage damage scores compared with Cre^−^ mice (Fig. 4c). In addition, the histomorphometric analysis also demonstrated significant reductions in the condylar cartilage area in β-catenin^Act^ mice (Fig. 4d). Moreover, we quantified the condylar cartilage thickness and found that the results were consistent with changes in the cartilage area (Fig. 4e). These results suggested that the conditional activation of β-catenin signalling led to TMJ chondrocyte hypertrophy and cartilage degeneration in 8-week-old mice, which was consistent with the results of our previous studies.^26^

**Fig. 5:**
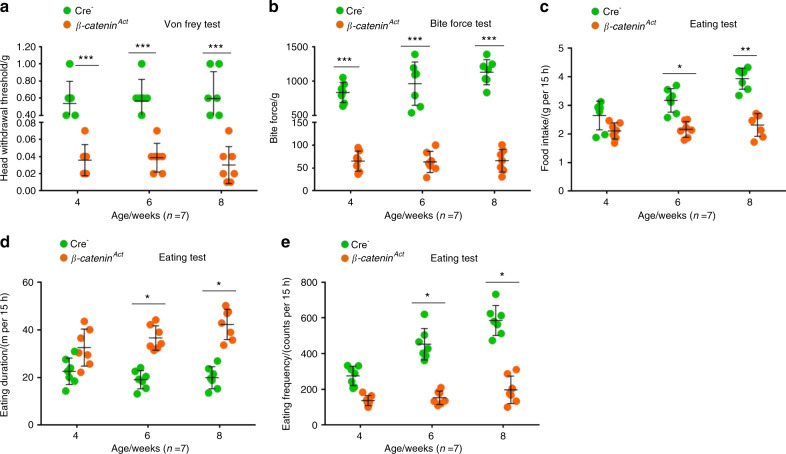
β-catenin^Act^ mice demonstrate increased mechanical allodynia in the TMJ region. **a** The mechanical allodynia threshold was measured using von Frey filaments at 4, 6, and 8 weeks of age (*n* = 7). **b** The bite force was measured using FlexiForce sensors at 4, 6, and 8 weeks of age (*n* = 7). **c–e** The food intake over 15 h was recorded, and the eating duration and frequency were assessed using LABORAS at 4, 6, and 8 weeks of age (*n* = 7). Statistical analysis was conducted using a two-way ANOVA, followed by the Tukey–Kramer post hoc test. *P < 0.05, **P < 0.01, ***P < 0.001

Before euthanizing the mice, we performed a series of behavioural tests to determine whether the conditional activation of β-catenin signalling increased mechanical allodynia in the TMJ region. The von Frey test results at 4, 6, and 8 weeks of age showed that the sensitivity of mechanical allodynia in the TMJ region significantly increased in the β-catenin^Act^ mice compared with the Cre^−^ mice (Fig. 5a), which was accompanied by a significant decrease in bite force (Fig. 5b). We also found that the 15-h food intake was significantly decreased in the 6- and 8-week-old β-catenin^Act^ mice compared with the Cre^−^ mice (Fig. 5c). Furthermore, the evaluation of 15-h spontaneous feeding behaviours using the Laboratory Animal Behaviour Observation Registration and Analysis System (LABORAS) demonstrated that both eating duration and frequency were significantly decreased in the 6- and 8-week-old β-catenin^Act^ mice compared with the Cre^−^ mice (Fig. 5d, e). These results suggested that the conditional activation of β-catenin signalling significantly increased mechanical allodynia in the TMJ region.

## Discussion

TMDs are clinically associated with severe local pain that is treatment refractory. Reliable experimental approaches for assessing changes in orofacial nociceptive behaviours in TMD mouse models are necessary to understand the pathological mechanisms underlying TMDs and to develop effective drug therapies. In this study, we explored several methods for assessing changes in nociceptive behaviours in two mouse models of TMD. The von Frey test has been implemented for the assessment of mechanical allodynia in rat TMD models.^7,29^ However, because mice are more disobedient and aggressive than rats, probing mechanical allodynia in the mouse orofacial region is difficult when attempting to use conditions similar to those used for rats, and few publications have reported successful assessments in mice. A previous study performed von Frey filament testing in mice that were restrained in tapered plastic bags, through which their heads protruded, which likely resulted in unstable measurement results because the animals were placed in an unnatural and stressful state.^30^ Another study used von Frey filaments to test a mouse model, but they only recorded the positive response of head withdrawal, rather than performing a quantitative threshold analysis.^31^ Therefore, the development of a stable experimental chamber that can house mice with TMD was necessary. In our experiments, we used a specially designed, solid plastic cage with a slight gap in its middle area (Fig. 1b), which allowed a filament to be inserted to probe the mouse TMJ region. The cage space was adjustable, and moderate space allowed the mouse to be unrestrained but motionless during the test session, avoiding the necessity of forcible restriction and immobilisation, which could result in a stress response that could confound the experimental outcomes. The successful identification of mechanical allodynia is critical for obtaining accurate testing results. The responses to mechanical stimuli during the von Frey test exhibited by mice appear to be more prompt, complicated, and variable than those in rats, including head shaking or brisk head withdrawal, ipsilateral forepaw swipes on the tested orofacial region, and attempts to attack the von Frey filament. Testers must be able to capture this information in a timely manner to make correct judgements. In addition, a wide variety of rat studies have reported the use of a series of calibrated von Frey filaments, with bending forces ranging from 8 mg to 112 g, to probe the facial site and determine an EF_50_ value, which is defined as the von Frey filament force necessary to induce a 50% response frequency.^9,32,33^ However, this method may not be optimal for mouse testing because it requires the serial application of more than a dozen filaments to the orofacial region, which can cause an uncomfortable, irritable reaction in mice, leading to potential injuries that could confound the experimental results. In this study, we used an ascending series of filaments, starting with the smallest filament, and terminated the test when the experimenter observed three positive responses out of five tests for a particular filament. Our results showed that the head withdrawal threshold significantly decreased in TMD mouse models, which was concordant with our hypothesis. Moreover, we found similar cartilage damage (Fig. S2) and no significant differences in mechanical allodynia (data not shown) between the left and right sides of the TMJ 3 weeks after the UAC operation, suggesting that the biomechanical load produced by the UAC prosthesis comparably affected both sides of the TMJ.

TMDs are also associated with mastication-related pain.^13,14^ When TMDs occur due to tissue injury in the relevant structures, mastication becomes painful, leading to decreased bite forces and the eventual reduction in food intake.^9,34,35^ It has been suggested that the measurement of bite force may represent a more clinically relevant feature of TMDs than the assessment of orofacial mechanical allodynia.^13,14^ Recently, several innovative methods were developed to directly test bite force.^13,14,36^ Dolan et al.^37^ used a dolognawmeter to quantify the gnawing functions of three mouse models of orofacial pain. In the current study, we employed a simple but more practical device to measure the bite force in mouse models of TMD: FlexiForce sensors and the Economical Load and Force (ELF) system, which has been demonstrated to be feasible for applications in 13 species of small mammals.^38^ Most commercially available sensors, such as sensors designed for humans, are too large to fit easily into the mouths of small mammals.^38^ The FlexiForce sensors can be small and thin, without losing sensitivity, which is advantageous when working with small animals, such as mice (Fig. 1c). Moreover, the utilisation of various coverings, such as thin steel, rubber, leather, and plastic, which are used to protect both the sensors and the teeth of the tested animals, did not have significant effects on the final testing results.^38^ In our study, a sensor with rubber pads was presented to the mouths of mice, and the force of a single bite was recorded. If the mouse bit several times in rapid succession, we selected the maximum value to represent the strongest bite force of the animal, as previously described.^38^

In addition, because the TMJ is the principal joint associated with mastication, the chewing patterns of TMD animals are likely to be altered, further influencing food intake and eating patterns.^16^ Our results showed that food intake decreased as the head withdrawal threshold decreased in both examined TMD mouse models, suggesting that food intake could serve as an indicator for orofacial nociceptive behaviours, as previously reported.^15–17^ Furthermore, the analysis of eating patterns has been suggested to be a noninvasive biological marker of TMJ inflammation and nociceptive behaviours.^15^ Therefore, we used the automated behavioural classification system LABORAS to detect changes in eating patterns in mouse models of TMD, including feeding duration and frequency. Our results indicated that both TMD mouse models showed significantly increased eating duration and decreased eating frequency, suggesting that nociceptive behaviour, likely caused by the movement of the TMJ during the chewing process, resulted in slowed and reduced eating. These findings were consistent with prior studies, in which eating patterns were tested using a photobeam system, in a rat orofacial nociceptive behavioural model caused by inflammation.^15,35^ However, TMDs may also affect other tissues, in addition to the TMJ, such as orofacial muscles and nerves, which could also lead to mastication dysfunction.^16–20^ Therefore, reduced bite force and changes in chewing behaviour might also reflect dysfunction of the jaw muscles and periodontal tissues,^21–24^ which requires further investigation. Indeed, alterations in bite force and eating behaviours might not be entirely attributable to TMD-associated changes in nociceptive behaviours. For example, non-painful TMDs have been observed in clinical practice, in which individuals experience joint dysfunction with no apparent symptoms of pain.

In recent years, mouse models have frequently been used to explore pathological mechanisms and to evaluate potential therapies for TMDs. In this study, to comprehensively verify the feasibility of assessment methods for nociceptive behaviours, we investigated both a mechanical stimulus-induced TMD mouse model and a genetic TMD mouse model, which are typically utilised in research. Our findings indicated that both the UAC-induced TMD mouse model and the β-catenin conditional activation mouse model experienced significant cartilage degradation. Both models showed significant increases in TMJ mechanical allodynia, reduced bite forces, and decreased spontaneous feeding behaviours compared with control mice. These results suggested that these assessment methods, including the von Frey filament test, the bite force test, and eating pattern analysis, could be used to analyse different TMD mouse models. To verify the reliability of these methods, other types of TMD models are also needed, including mechanical stimulus-induced models, in which specific appliances have been employed to develop abnormal occlusal conditions,^39,40^ the noxious stimulus model by orofacial formalin injection,^41^ the TMJ inflammation mouse model, which is induced by the injection of complete Freund’s adjuvant,^42,43^ and genetic mouse models.^13,44^ Moreover, these methods should be further optimised in future experiments.

In this study, we used male mice for the behavioural assessments because tamoxifen is known to have variable effects in females, and oestrogen levels can influence TMD-associated nociceptive behaviours,^45,46^ which could have interfered with our results and conclusions. However, feasible nociceptive behaviour assessment approaches for female mice with TMDs should be developed in the future because TMDs have been reported to be more prevalent among women than men.^47,48^

In summary, we explored three methods, the von Frey filament test, the bite force test, and eating behavioural evaluations, for the assessment of changes in nociceptive behaviours in two TMD mouse models. These results demonstrated that mechanical allodynia in the TMJ region significantly increased in both the UAC-induced TMD mouse model and the β-catenin conditional activation mouse model. Our findings suggested that these behavioural assessment methods are feasible and reliable for the detection of sensitivities of nociceptive behaviours and could play essential roles in the study of nociceptive behaviours in various TMD mouse models.

## Materials and methods

### Animals and the UAC model

The Institutional Animal Care and Use Committee (IACUC) of the Rush University Medical Center approved the animal protocols used in this study. All experimental methods and procedures were performed according to the approved guidelines and complied with all relevant ethical regulations for animal testing research. The experimental flow chart is shown in Fig. 1a.

Twenty 6-week-old male C57BL/6J mice were randomly divided into two groups (*n* = 10 in each group). TMD was induced by mis-occlusion, using UAC, as previously described.^25^ Briefly, anaesthesia was administered by intraperitoneal (i.p.) injection of 1.2% tribromoethanol (Sigma-Aldrich, #T48402) at 240 mg·kg^−1^ body weight. In the experimental group, the left maxillary and mandibular incisors of the mice were bonded with metal tubes made from syringe needles (#18-gauge, inner diameter = 0.61 mm, thickness = 0.3 mm). The maxillary tubes were 2.0 mm long to fit the maxillary incisors, and the mandibular tubes were curved to form a 135°, labially inclined, occlusal plate. The tubes were carefully bonded with zinc phosphate cement (Dental Health Products Inc., USA). The mice were then fitted with the UAC prosthesis (Fig. S1), forcing the TMJ to bear extra loading during incising and chewing to promote TMJ degeneration. In the control group, the mice underwent the same procedure, but the metal tubes were immediately removed to prevent the incisors from experiencing excessive mechanical loading. The mice were fed a normal diet, and body weights were recorded every other day. The TMJs of all mice were collected three weeks after the UAC operation.

Based on our previous studies,^26^ β-catenin^Act^ mice were generated by crossing Agc1-CreER^T2^ mice with β-catenin(ex3)^flox/flox^ mice. Our previous studies demonstrated that Agc1-CreER^T2^ mice could efficiently drive Cre expression in TMJ cells.^26,49^ Cre-negative littermates were used as the control group. β-catenin^Act^ mice and Cre-negative littermates (male) were administered tamoxifen (Sigma, St. Louis, MO, USA) by i.p. injection (1 mg per 10 g body weight for five consecutive days) at 2 weeks of age. We demonstrated in previous studies that this dosing regimen could efficiently induce Cre-mediated recombination and activate β-catenin signalling in condylar chondrocytes.^26^ Condylar cartilage samples were harvested from both groups of mice at 8 weeks of age (*n* = 7 in each group). The von Frey test, the bite force test, and the eating tests in both groups of mice at 4, 6, and 8 weeks of age (*n* = 7 in each group) were conducted in a single-blind manner.

### Histology analysis

After euthanasia, the mouse skulls were dissected, and samples containing TMJ tissue were fixed in 10% neutral-buffered formalin (VWR, Radnor, PA, USA) for 3 days, followed by decalcification with formic acid (Decal Chemical Corp., Suffern, NY, USA) for 14 days. Samples were processed and embedded in paraffin, and 3-µm-thick mid-sagittal sections were stained with Alcian blue and haematoxylin and eosin for morphologic analysis. The histology analysis was performed by one investigator who was blinded to the experimental conditions. The severity of the OA-like phenotype was analysed according to the OARSI scoring system using three-level sections of the condylar cartilage, as previously described^50,51^ (OARSI scoring system: 0 = normal; 1 = small fibrillations; 2 = superficial layer clefts; 3 = clefts/erosion in <25% of the cartilage surface; 4 = clefts/erosion in 25%–50% of the cartilage surface; 5 = clefts/erosion in 50–75% of the cartilage surface; and 6 = clefts/erosion in >75% of the cartilage surface). We also quantified the cartilage area using OsteoMeasure software (OsteoMetrics, Inc., Atlanta, GA, USA) to measure the Alcian blue-positive area, which assesses changes in condylar histology. Condyle cartilage thickness was also measured using the Leica DFC490 system (Leica, Wetzlar, Germany), as previously reported.^52,53^ Briefly, four bold lines were used to divide the condylar cartilage into anterior (AP), central (CP), and posterior (PP) thirds, and three slender lines further divided the central and posterior thirds into four smaller portions, as shown in Fig. S3. The cartilage thicknesses of the anterior, central, and posterior thirds were measured as the average lengths of the three slender lines in each corresponding third.^53^

### Behavioural assessments

#### Mechanical allodynia test

The mechanical response threshold of escape behaviour was measured in mice using an ascending, calibrated series of von Frey filaments (North Coast Medical Inc., CA, USA), starting with the smallest filament, with a bending force of 0.008 *g* (filament size 1.65). Our method was adapted from the ‘rat von Frey test method’, which has been previously published.^7,54,55^ Briefly, before the test, the mice were housed in a special plastic cage, with a slight gap in the middle, for 30 min on 3 consecutive days for acclimation. Then, the orofacial areas directly overlaying the TMJ were carefully shaved under anaesthesia, avoiding the whiskers, 1 day before the starting date of the testing session. On the experimental procedure day, after adapting to the cage and environment for 30 min, the mice were calm, and attention was focused towards the front. The filaments were applied perpendicular to the bilateral orofacial skin and depressed slowly until bending occurred, as shown in Fig. 1b. Each filament was tested five times, with a few seconds between each test. If head escape behaviours were observed at least three times, the mouse was considered to be responsive to that filament, and the force of that filament was recorded; otherwise, the test was regarded as a negative response, and the next larger filament was applied. The mean filament size for both sides was calculated as the final force value. Positive responses were defined as follows: (1) slowly turning the head away or briskly moving the head backward; (2) spontaneously and rapidly shaking the head; (3) a single or multiple ipsilateral forepaw swipes to the stimulated orofacial area; and (4) actively attacking the stimulus object, making biting and grabbing movements (Supplementary Videos 1 and 2). For β-catenin^Act^ mice, mechanical allodynia tests were performed at 4, 6, and 8 weeks of age (*n* = 7), whereas for UAC mice, mechanical allodynia was tested in 7-, 8-, and 9-week-old mice (1–3 weeks after the UAC operation, respectively) (*n* = 10).

#### Bite force test

To measure bite force, we used FlexiForce sensors (B201-L-8) and the ELF system, manufactured by Tekscan Inc. (South Boston, MA, USA; Fig. 1c). USB-interface data acquisition handling was used to connect the sensor with a laptop (Dell, Inspiron 15-7535), on which the complete ELF system software was installed. Prior to the test, the mice were housed in a special plastic cage with a slight gap for 30 min to allow them to acclimate and calm down. The sensor, which was adhered to two rubber pads, was calibrated for linearity by loading a series of calibration weights, ranging from 1 to 1000 *g*. Based on the standard protocol for the bite force test,^38^ we measured the animal for 1 min without pain stimulation, and consecutively recorded raw data were analysed (Fig. 1d, e). The same test was performed three times, with 3-min intervals between each trial. The maximum bite force (in grams) of each animal was recorded for each trial, and then all trials were averaged. For β-catenin^Act^ mice, the bite force tests were completed at 4, 6, and 8 weeks of age (*n* = 7), whereas for UAC mice, the bite force was only measured once, 3 weeks after the procedure through unloading the UAC prosthesis under anaesthesia (*n* = 10).

#### Eating test

Spontaneous behaviour was assessed using LABORAS (Metris, Netherlands), which can detect vibrations evoked by the movement of a single rodent in a cage. This pattern-recognition software can classify and quantify behaviours, including grooming, mobility, climbing, immobility, and feeding.^56,57^ In this study, the mice were acclimatized to the equipment on three occasions before measurement. On the experimental procedure day, the animals were weighed and housed on the test platform for 15 h, from 18:00 to 09:00 the following day. When the animals ate food pellets while standing upright, gripped the bars of the food hopper, or ate pieces of food pellets from the floor, the software recorded these behaviours and analysed the time and frequency of eating. For example, when the mouse stands upright to eat food pellets in the hopper, this action was recorded as one incident for the eating frequency measure, regardless of how many chewing cycles the mouse engaged in. The eating duration was also recorded, as was the amount of food intake. For the β-catenin^Act^ mice, the eating tests were completed at 4, 6, and 8 weeks of age (*n* = 7), whereas for the UAC mice, we measured the eating behaviours in 7-, 8-, and 9-week-old mice (1–3 weeks after the UAC operation, respectively) (*n* = 10).

### Statistical analysis

All data are presented as the mean ± standard deviation (s.d.), as indicated in the figure legends. Statistical analyses were completed using GraphPad Prism software. Unpaired Student’s *t* test for comparisons between two groups, one-way or two-way analysis of variance (ANOVA) for comparisons among multiple groups with one or two variables, were used, followed by the Tukey–Kramer post hoc test. *P* < 0.05 was considered significant.

## Supplementary information

Suppl Figs

Suppl video 1

Suppl video 2
