# Supplementary material for: Nociceptive behavioural assessments in mouse models of temporomandibular joint disorders
Source: Int J Oral Sci. 2020 Sep 29;12:26. doi: 10.1038/s41368-020-00095-0 (PMC7522224; doi:10.1038/s41368-020-00095-0)
Supplement: Supplementary file 1 — Suppl Figs [file 41368_2020_95_MOESM1_ESM.pdf]

Nociceptive Behavioral Assessments in Mouse Models of Temporomandibular Joint Disorders

Jun Li[1], Kaige Ma[1], Dan Yi[2], Chundo Oh[1] and Di Chen[1,2.]

[1]Department of Orthopedic Surgery, Rush University Medical Center, Chicago, IL 60612, USA,

[2]Research Center for Human Tissues and Organs Degeneration, Shenzhen Institutes ofAdvanced Technology, ChineseAcademy of Sciences, Shenzhen 518055, China

Lateral View Frontal View

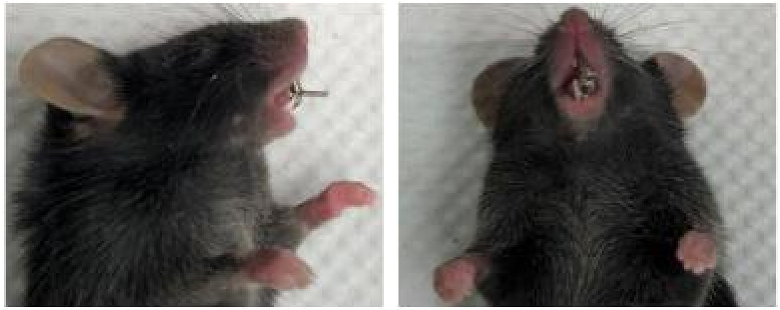

C57BL/6 mice

Figure S1. The representative frontal and lateral view of anterior teeth malocclusion with UAC prosthesis.

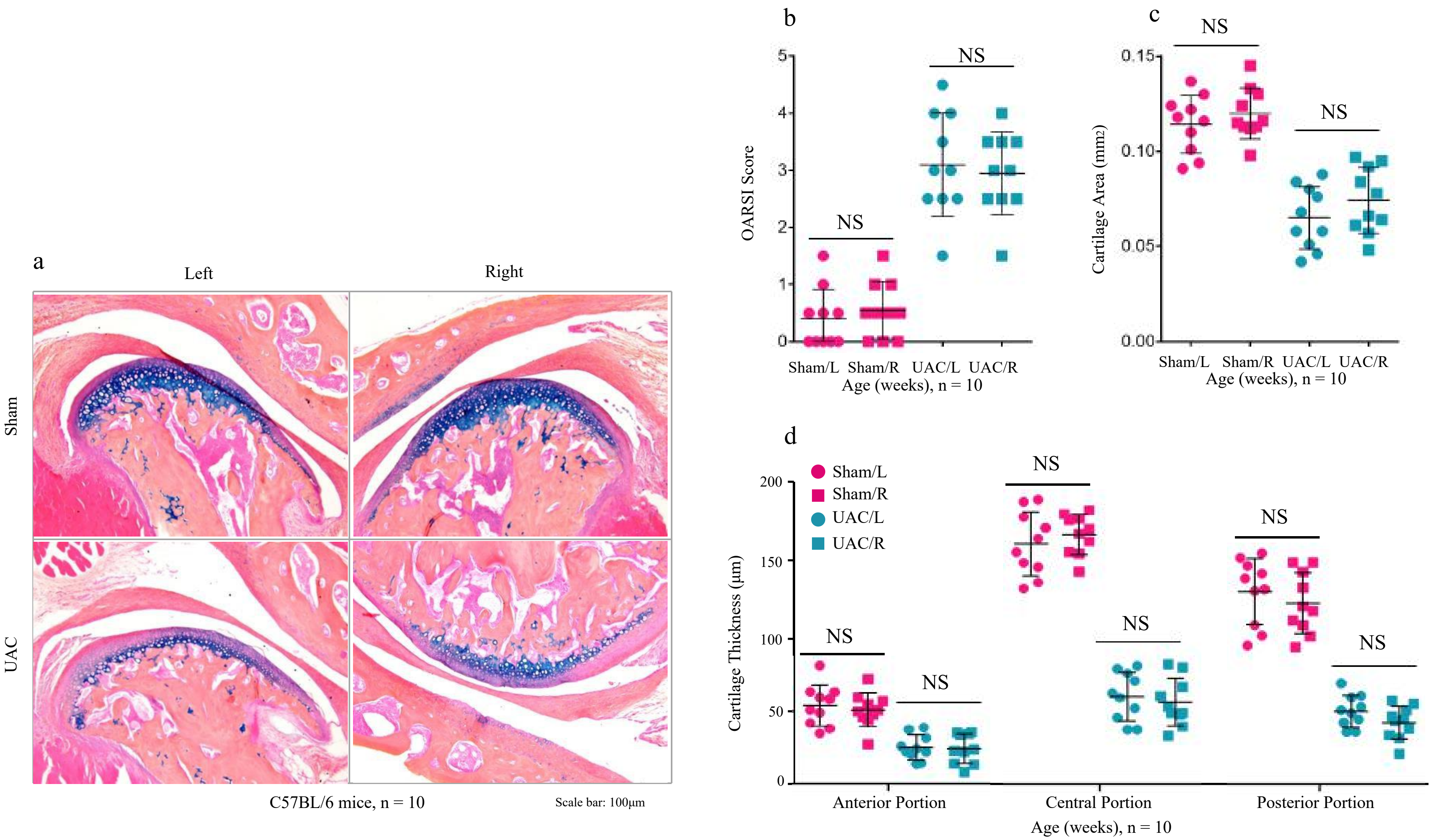

Figure S2. Compare the cartilage degeneration in both sides of mandibular condyle 3 weeks after UAC operation. No significant differences between left and right condyle cartilage in sham or UAC group were found in Alcian blue/hematoxylin staining (a), OARSI score (b), cartilage area (c), and cartilage thickness (d). Sham/L: Sham/Left; Sham/R: Sham/Right; UAC/L: UAC/Left; UAC/R: UAC/Right.

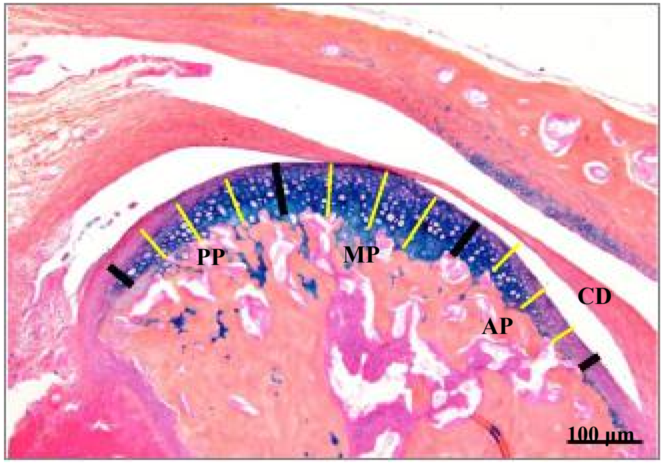

Figure S3. The representative central sagittal Alcian blue/Hematoxylin staining section of TMJ 3 weeks after UAC operation. CD: Cartilage disc; AP: Anterior portion; MP: Middle portion; PP: Posterior portion. The anterior, central and posterior third cartilage thickness was measured as the average length of the three lines in the corresponding thirds.
